# Sex Ratio of Small Hive Beetles: The Role of Pupation and Adult Longevity

**DOI:** 10.3390/insects10050133

**Published:** 2019-05-07

**Authors:** Anna Papach, Jérémy Gonthier, Geoffrey R. Williams, Peter Neumann

**Affiliations:** 1 Institute of Bee Health, Vetsuisse Faculty, University of Bern, 3097 Bern, Switzerland; jeremy.gonthier@students.unibe.ch (J.G.); peter.neumann@vetsuisse.unibe.ch (P.N.); 2 Department of Entomology & Plant Pathology, Auburn University, Auburn, AL 36849, USA; grw0010@auburn.edu; 3 Swiss Bee Research Centre, Agroscope, 3097 Bern, Switzerland

**Keywords:** small hive beetle, pupation, sex ratio, longevity

## Abstract

The sex ratio of sexually reproducing animal species tends to be 1:1, which is known as Fisher’s principle. However, differential mortality and intraspecific competition during pupation can result in a biased adult sex ratio in insects. The female-biased sex ratio of small hive beetles (SHBs) is known from both laboratory and field studies, but the underlying reasons are not well understood. Here, we used laboratory mass and individual pupation to test if differential mortality between sexes and/or intraspecific interactions can explain this sex ratio. The data show a significant female-biased adult sex ratio in both mass and individual rearing, even when assuming that all dead individuals were males. Our results therefore suggest that neither differential mortality during pupation nor intraspecific interactions are likely to explain the female-biased sex ratio of freshly emerged adult SHBs. We regard it as more likely that either competition during the larval feeding stage or genetic mechanisms are involved. In addition, we compared our data with previously published data on the sex ratio of both freshly emerged and field-collected SHBs to investigate possible gender differences in adult longevity. The data show a significantly greater female bias in the sex ratio upon emergence, compared to field-collected SHBs, suggesting that adult females have a shorter longevity.

## 1. Introduction

The ratio of adult females to males is a key parameter for the biology of any sexually reproducing animal species [1]. In most species, the sex ratio tends to be 1:1, which is known as Fisher’s principle. However, there are several insect and mite species that have a female-biased (e.g., fig wasps [2]) or male-biased sex ratio (e.g., honeybees *Apis* sp. [3]) due to a range of different factors (different costs, polyandry, etc. [4]). For small hive beetles (SHBs), *Aethina tumida* Murray (Coleoptera: Nitidulidae), several studies suggest a female-biased sex ratio in the laboratory [5,6,7] and in the field [8,9]. SHB is a parasite and scavenger of honeybee colonies, *Apis mellifera*. It is endemic to sub-Saharan Africa [10] and has spread to all habitable continents [11,12,13,14,15]. Within honeybee colonies, adult SHBs feed on honey, pollen, bee brood, dead/live adult bees, and/or are fed trophallactically by the hosts [15]. The adult SHBs mate and oviposit in the nests. Then, the emerging larvae feed on all available food sources, thereby often destroying the entire nest, and finally leave the colony to pupate in nearby soil [15]. In its endemic range, the SHB is usually considered a minor pest of honeybee colonies [16]. However, in the new ranges, SHBs can considerably harm local honeybee populations and, possibly, other bee species [15,16,17].

Even though the female bias in the sex ratio of SHBs has long been known, the underlying reasons are not well understood. Competition between larvae for resources [18,19], differential mortality during pupation [20,21], adult longevity in the field, and/or intraspecific competition [22,23] might contribute. Finally, a genetically determined primary sex ratio [24] and/or differential mortality of adults in the field [25] may be involved. Indeed, preliminary observations suggest that adult SHBs may have a reduced longevity, possibly due to costs associated with oviposition [15].

Here, we take advantage of mass pupation and individual pupation (thereby excluding any interspecific interactions) of SHBs in the laboratory to test whether differential mortality and/or intraspecific competition between sexes are involved in the observed sex ratio of freshly emerged adults. Moreover, we compared our data with previously published data on the sex ratio of freshly emerged and field-collected SHBs of random ages to investigate possible gender differences in adult longevity. Given that the sex ratios of freshly emerged and field-collected adults differ, this would support differential adult longevity in this insect species.

## 2. Materials and Methods

### 2.1. Pupation Experiments

Experiments were conducted at Auburn University, AL, USA, from May to July, 2018. Adult SHBs were collected from naturally infested local honey bee colonies, sexed [26], and used to initiate laboratory rearing [27]. In brief, 13 randomly chosen SHB females were individually placed in 473 mL glass jars with punctured lids, provided with pollen and honey paste (2:1), and oviposition sites (two microscope slides separated with a half of a cover slip on each end and taped together), and then incubated at 25 °C, 80% RH. The jars were checked daily, and the larvae were fed ad libitum until they reached the post-feeding wandering stage.

#### 2.1.1. Individual Pupation

**Figure 1:**
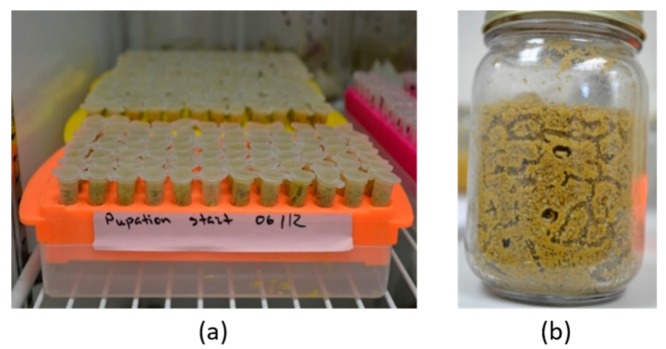
Small hive beetle (SHB) individual and mass pupation approaches: (**a**) tube trays containing Eppendorf tubes with individually pupating larvae; (**b**) a pupation jar containing 30 pupating larvae.

Standard Eppendorf tubes (1.5 mL, *n* = 513) with punctured lids for ventilation were 75% filled with autoclaved sandy soil that was ~10% moisture by mass. Then, a single wandering larva was gently transferred onto the soil of each tube with a fine paintbrush and the tubes incubated at 25 °C and 80% RH until adult emergence (Figure 1). Any larva that was still found on the soil surface 12 hours later was replaced with another larva. After all larvae were buried, the tubes were visually checked every four days and a drop of water was added each time. All emerging beetles were sexed [26].

#### 2.1.2. Mass Pupation

Wandering larvae (*n* = 30 each) were gently transferred with a fine paint brush into 13 pupation jars (473 mL), and 75% filled with autoclaved sandy soil that was ~10% moisture by mass (Figure 1). All jars were incubated as described above until adult emergence. As adult SHBs tend to congregate under the soil surface in the pupation chambers [6], the content of each pupation jar was sifted to collect the mature adults after the emergence of the first beetle in that jar. All adult beetles were sexed upon emergence [26].

### 2.2. Sex Ratio Field vs. Laboratory

Previously published laboratory (Republic of South Africa (RSA): [5,6]) and field (Australia, RSA and USA: [8,9]) data were combined with the present laboratory data, and used to test whether there are differences in the sex ratio between newly emerged and field-collected adults of random ages. The laboratory data for freshly emerged individuals were chosen because the SHBs were reared under nearly identical conditions. The field data were obtained by collecting and sexing beetles of unknown ages from naturally infested local honeybee colonies [8,9].

### 2.3. Statistical Analyses

The numbers of emerged adult SHBs were compared between mass and individual pupation approaches using a χ^2^ test of independence. A Wilcoxon test was used to test the differences of pupation duration between the two approaches. The sex ratio of emerging adults was compared to a 1:1 ratio using a χ^2^ goodness of fit test. To see if there were differences in the sex ratio between mass and individual pupation approaches, the numbers of emerging males and females were compared using a χ^2^ test of independence. To compare the sex ratios of the field collected beetles with newly emerged beetles, we pooled the available laboratory [5,6] and field data [8,9] with our own data, and compared it using a χ^2^ test of independence. All calculations were performed using R version 3.5.1 (R Core Team, Vienna, Austria).

## 3. Results

### 3.1. Pupation Experiments

Females started ovipositing one week after the start of the experiment. From day 12 to 17, larvae reached the wandering stage. The first adult beetles emerged after 17 days in the mass rearing approach up to day 21, and up to day 26 in the individual rearing approach. There were no differences in pupation duration between the mass and individual rearing approaches (W = 56,884; *p* = 0.46). 

A total of 391 of the 394 wandering larvae that were introduced into the mass pupation jars successfully pupated and emerged as adults, resulting in a 99% pupation success rate. In the individual rearing approach, 433 of the 513 wandering larvae that were introduced into the Eppendorf tubes emerged as adults, resulting in an 84% pupation success rate. There was a significantly higher pupation success rate (χ^2^ = 59.7, *p <* 0.01) in the mass rearing approach compared to the individual rearing approach. A fungus was spotted in two tubes with dead larvae.

A significantly female-biased sex ratio was observed in the emerging beetles, both reared with the mass approach (χ^2^ = 48.3, *p <* 0.01) and with the individual approach (χ^2^ = 25.5, *p* < 0.01). The sex ratio of emerging beetles did not significantly differ between rearing approaches (χ^2^ *=* 2.47, *p* = 0.12). The emerging beetles had a female-biased sex ratio even when all dead beetles were assumed to be males (χ^2^ = 27.6, *p* < 0.01).

### 3.2. Sex Ratio Field vs. Laboratory

The ratio of females to males of newly emerged laboratory-reared beetles ([5]—1.53; [6]—1.59, present study—1.58) was even more female-biased (χ^2^ = 18.692, *p* < 0.01) compared to the field-collected beetles ([8]—0.93; [9]: RSA—1.31, Australia—1.51). 

## 4. Discussion

Our data confirm the female-biased sex ratio of newly emerged SHBs [6,7,8] and further suggest that neither differential mortality nor any intraspecific interactions during pupation are involved in any way. The sex ratio of those emerged SHBs remained female-biased, even when assuming that all the individuals that died during pupation were males. Moreover, there were no differences between mass and individual rearing with respect to sex ratio. However, pupation success in the mass rearing approach was significantly higher than in the individual approach, probably due to higher evaporation rates in the Eppendorf reaction tubes leading to suboptimal soil humidity (see [15]).

The previously reported female-biased SHB sex ratio of newly emerged SHBs in the laboratory has two remarkable exceptions [18,28]. These studies reported no deviations from a 1:1 sex ratio in freshly emerged laboratory reared SHBs that were fed according to unusual diets (e.g., fruits), except for the groups that were fed on pollen and brood comb and that also had the highest number of larvae [18]. There was a significantly female-biased sex ratio only in the pollen and brood diets, thereby supporting the idea that in a situation of limited resources, feeding female SHB larvae may outcompete males [18]. Indeed, adult female SHBs tend to be bigger than males [15,26], which may suggest a possible competitive advantage of the presumably larger larvae of the females. Finally, there is the possibility that SHBs already have a female-biased sex ratio at the egg stage, due to having genetic mechanisms similar to those in *Drosophila* [24].

Our results further show that the sex ratio of freshly emerged SHBs is significantly more female-biased compared to field-collected SHBs of various ages. This suggests that females might have a shorter/decreased longevity compared to males, possibly due to frequent oviposition, as earlier suggested [15].

## 5. Conclusions

Our data cannot explain the female-biased sex ratio of emerging adult SHBs, but do suggest that pupation is very unlikely to contribute. Competition between larvae and the primary sex ratio should be considered in follow-up studies. Furthermore, our analyses support previous preliminary observations that the longevity of adult females is reduced, possibly due to costs associated with oviposition [15]. This calls for more efforts to investigate the possible mechanisms responsible for sex-specific differences in adult longevity, including the costs of oviposition for this species.
